# CircRNA expression profile of bovine placentas in late gestation with aberrant SCNT fetus

**DOI:** 10.1002/jcla.22918

**Published:** 2019-05-26

**Authors:** Xiaohu Su, Guangqi Gao, Shenyuan Wang, Guanghua Su, Zhong Zheng, Jiaqi Zhang, Lidong Han, Yu Ling, Xiuying Wang, Guangpeng Li, Li Zhang

**Affiliations:** ^1^ The State Key Laboratory of Reproductive Regulation and Breeding of Grassland Livestock Inner Mongolia University Hohhot China; ^2^ Key Laboratory of Gene Engineering of the Ministry of Education, Guangzhou Key Laboratory of Healthy Aging Research and State Key Laboratory of Biocontrol, SYSU‐BCM Joint Research Center, School of Life Sciences Sun Yat‐sen University Guangzhou China; ^3^ Key Laboratory of Biological Manufacturing of Inner Mongolia Autonomous Region, College of Life Sciences Inner Mongolia Agricultural University Hohhot China; ^4^ State Key Laboratory of Stem Cell and Reproductive Biology Institute of Zoology, Chinese Academy of Sciences Beijing China; ^5^ Inner Mongolia Radio and TV University Hohhot China

**Keywords:** aberrant development, bovine, circular RNAs, placenta, somatic cell nuclear transfer

## Abstract

**Backgrounds:**

One of the limitations of somatic cell nuclear transfer (SCNT) strategy to generate genetically modified offspring is the low birth rate. Placental dysfunction is one of the causes of abortion. Circular RNA (circRNA) is noncoding RNA which functions as microRNA (miRNA) sponges in biological processes.

**Methods:**

Two aberrant pregnant placenta (aberrant group, AG) and three normal pregnant placenta (normal group, NG) during late gestation (180‐210 days) with bovine SCNT fetus were collected for high‐throughput sequencing and analyzed. The host genes of differentially expressed (DE) circRNAs were predicted. And the microRNAs (miRNAs) which could interact with DE circRNAs were analyzed. Then, the expressional level of partial DE circRNAs and corresponding host genes was verified through qRT‐PCR. At last, the function of host genes was analyzed through Gene Ontology (GO) and Kyoto Encyclopedia of Genes and Genomes (KEGG).

**Results:**

Altogether 123 differentially expressed circRNAs between two groups were identified, which were found related to 60 host genes and 32 miRNAs. The top 10 upregulated circRNAs were bta_circ_0012985, bta_circ_0013071, bta_circ_0013074, bta_circ_0016024, bta_circ_0013068, bta_circ_0008816, bta_circ_0012982, bta_circ_0013072, bta_circ_0019285, and bta_circ_0013067. The top 10 downregulated circRNAs were bta_circ_0024234, bta_circ_0017528, bta_circ_0008077, bta_circ_0003222, bta_circ_0007500, bta_circ_0020328, bta_circ_0011001, bta_circ_0016364, bta_circ_0008839, and bta_circ_0016049. The qRT‐PCR results showed consistent trend with sequencing analysis result, while host genes had no statistic difference. The GO and KEGG analyses of the host genes suggested that abnormal circRNA expression may play multiple roles in placental structure and dysfunction.

**Conclusion:**

The abnormal circRNA expression may be one of reasons of placental dysfunction, leads to abortion of bovine SCNT fetus.

## 1 INTRODUCTION

During the past two decades, tremendous progress has been achieved in animal cloning since the birth of Dolly. One major breakthrough in the field, somatic cell nuclear transfer (SCNT), has given birth to a barnyard of livestock animals, including cattle, pig, sheep, and goat.^1^ Combined with gene editing technology, this technique had proven valid in developing genetically modified livestock. However, one of the bottlenecks of SCNT is low birth rate. Only 6% of transferred cloned embryos result healthy offspring in cattle.^2^ According to our previous research, the survival rate of genetically modified cloned cattle was below 5%. Incomplete reprogramming is amenable to the developmental failure of cloned embryo.^3^, ^4^ Except for the fetal aberrant development, placental dysfunction, such as reduced vascularization, placentomegaly, hypoplasia of trophoblastic epithelium, and altered basement membrane, was another cause to lead pregnancy losses.^2^, ^4^ We found that a fairly large number of cloned cattle aborted during late gestation (180‐210 days). The abnormal pregnant recipient showed engorged uterus and enlarged umbilical vessels. Coincidentally, a equine clone research depicted similar symptoms.^5^ It indicates that this is a relative common abnormality during SCNT fetal pregnancy, while the causes are ambiguous.

Placenta is a circular organ which temporarily exists in placental mammals during gestation. It not only supplies the space for fetus with protection and nutrition metabolism, but also secretes multiple growth factors and hormones to maintain gestation. In addition, it is the only pathway to connect the mother and the fetus. Placental research by RNA‐seq for abortion and aberrant pregnancy in livestock mainly focused on early gestation or postnatal.^6^, ^7^, ^8^, ^9^ little about which However, few studies have looked at the placenta during the third trimester, when large quantity of SCNT fetal abortion occur.

Circular RNAs (circRNAs) were first discovered in RNA viruses as early as the 1970s.^10^ It formed as covalently closed loop structures with neither 5′‐3′ polarities nor polyadenylated tails and more stable than linear RNA.^11^ Serious reports showed that circRNAs could function as miRNA sponges, regulate alternative splicing, and modulate the expression of mRNAs.^12^, ^13^, ^14^, ^15^ The different types of RNAs serve different roles and form a network called the competing endogenous RNAs (ceRNAs).^16^ Like other noncoding RNAs, circRNAs have been associated with a particular role in biological development and disease initiation and progression.^17^ They have been found implicated with various cancers, including colorectal, lung, and cervical cancer.^18^, ^19^, ^20^ Hitherto noticed features of circRNA are mainly based on evidence gathered from human, and studies on other species are insufficient.^21^, ^22^, ^23^, ^24^

This study aims to explore the multiple factors that potentially lead to high abortion frequency exist in SCNT fetus generation during late gestation. To this end, we collected two aberrant pregnant placenta (abnormal group, AG) and three normal pregnant placenta (normal group, NG) at late gestation (180‐210 days) of bovine SCNT fetus. We acquired five bovine late gestational placental circRNA expression profiles and analyzed its differentiation. We sought to uncover the mechanism associated with this phenomenon. The discovery may provide a new insight for SCNT fetal aberrant development and improve the SCNT efficiency.

## 2 MATERIALS AND METHODS

### 2.1 Ethics statement

All experimental procedures and sample collections were conducted in accordance with the Regulations for the Administration of Affairs Concerning Experimental Animals (Ministry of Science and Technology, China; revised in August 2011) and were approved by the Institutional Animal Care and Use Committee of Inner Mongolia University, Hohhot, China.

### 2.2 Sample information and collection

Cloned embryo, embryo transfer, and recipient cow experimental work were supplied by Inner Mongolia University. Briefly, the donor cell was fetal skin fibroblast. The recipients were 2‐5 years. The procedure was followed as Wu et al.^25^

A total of five late pregnant cows were used in the present study from two groups, that is, the aberrant pregnant cows (aberrant group, AG: n = 2) and normal pregnant cows (Normal group, NG: n = 3). All of the selected cows were at late pregnancy stage (180‐210 days). After the pregnant cows were slaughtered, the placenta was rapidly harvested and immediately frozen in liquid nitrogen and stored for use toward the subsequent generation of circle RNA libraries.

### 2.3 RNA preparation

The total RNA was extracted using TRIzol™ reagent (Invitrogen) following the manufacturer's procedure.^26^ Briefly, 50‐100 mg of tissues was lysed by 1 mL of TRIzol™ reagent. 0.2 mL of chloroform per 1 mL of TRIzol™ Reagent was added after 5 minutes of incubation. Then, the samples were centrifuged for 15 minutes at 12 000 *g* at 4°C after 2‐3 minutes incubation. The mixture separated into a lower red phenol‐chloroform, and interphase, and a colorless upper aqueous phase. The RNA was contained in the aqueous phase. The aqueous phase was transferred to a new tube and added 0.5 mL of isopropanol. After incubation of 10 minutes, centrifuge for 10 minutes at 12 000 *g* at 4°C. The supernatant was discarded and 1 mL of 75% ethanol was added to wash RNA. Centrifuge for 5 minutes at 7500 *g* at 4°C. The supernatant was discarded and air‐dried the RNA pellet for 5‐10 minutes. At last, the RNA was resuspended in 20‐50 µL of RNase‐free water. The quantity and purity of total RNA were analyzed using the Bioanalyzer 2100 (Agilent) with RIN number >7.0.

### 2.4 Library synthesis and high‐throughput sequencing

Approximately 3 µg of total RNA was used to prepare the circRNA library. Ribo‐Zero™ Gold Kits were used to degrade rRNA, and linear RNA was degraded by RNase R. Then, RNA libraries were generated according to the protocol outlined for NEBNext Ultra Directional RNA Library Prep Kit for Illumina (NEB). We then performed the single‐end sequencing on an Illumina Hiseq2500 at the ANOROAD GENOME Co., Ltd. (Beijing, CN) following the vendor's recommended protocol.

### 2.5 Differentially expressed circRNA analyses

The differentially expressed circRNAs between AG and NG were calculated by edge R using the likelihood ratio test (LRT) based on generalized linear model which estimates probability distributions according to mean‐variance relationship of each gene.^27^ Only transcripts with expression greater than 0.1 count per million (CPM) in at least one samples were selected for differential testing. Transcripts with *P* < 0.05 and |log2 ratio| ≥ 1 were considered differentially expressed.

### 2.6 Validation of differentially expressed circRNAs through qRT‐PCR

Eight differentially expressed circRNAs and relative host genes were selected for validation. Total RNA was extracted as previous. PrimeScript™ RT reagent kit (TAKARA) was used to cDNA synthesis, and only random 6‐mers were added. TB Green™ Premix Ex Taq™ II was used to qRT‐PCR. The procedure was followed as the manufacturer's instruction book. The primer sequences were listed at supplemental Table S1. The *GAPDH* was used as reference gene. The relative expression level of each circRNA and host gene was calculated using the 2^−ΔΔCt^ method. The data are indicated as the means ± SE (n = 3). The significance of the expression in two samples was calculated using a two sample *t* test in SPSS statistical software (Version17.0), whose difference was considered as significant when *P* < 0.05.

### 2.7 Functional enrichment analysis of host genes of differentially expressed circRNAs

The enrichment analyses of KEGG (Kyoto Encyclopedia of Genes and Genomes) and GO (Gene Ontology) were performed using DAVID (The Database for Annotation, Visualization, and Integrated Discovery) with the default parameters.

### 2.8 Target miRNAs of differentially expressed circRNA prediction and co‐expression network analysis

The target miRNAs of differentially expressed circRNAs were evaluated using miRanda (3.3a), investigating only perfect seed matching without gap of wobble pairing (“strict” parameter).^28^ A hit between any expressed miRNA (including the new predicted miRNA) and a target circRNA was considered for a miRanda score of 140 or higher, corresponding to at least a perfect seed match.

## 3 RESULTS

### 3.1 Characteristics of bovine placental circRNA expression pattern

In this study, we analyzed two aberrant pregnant placenta (AG) and three normal pregnant placenta (NG) at late gestation (180‐210 days) with bovine SCNT fetus. To study the general characteristics of all circRNAs in bovine placenta, we performed a preliminary analysis of all these sequencing results. A total of 12 454 circRNAs were evaluated, 6161 and 10 544 circRNAs of AG and NG, respectively.

**Figure 1:**
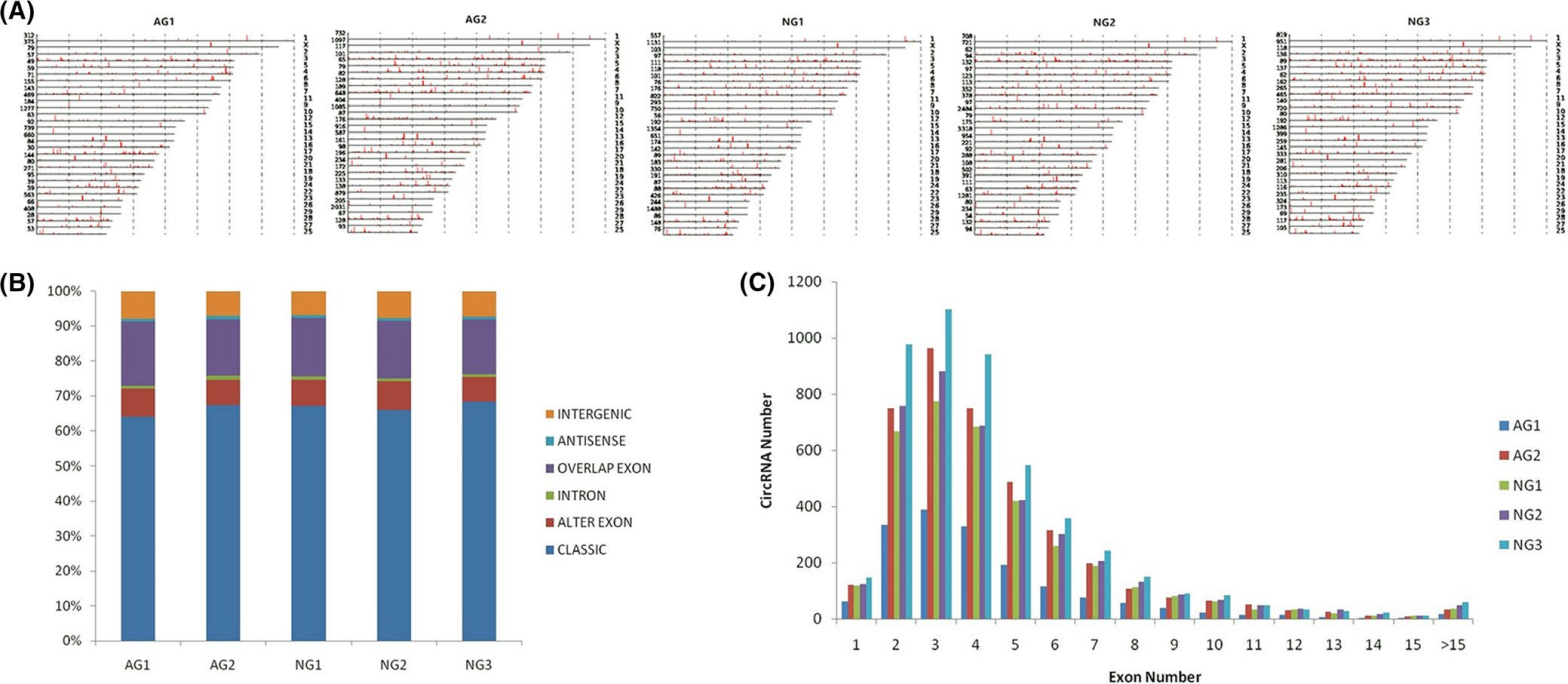
Characteristics of genomic location and classification of circRNAs expressed in bovine late gestational placenta. A, The chromosome distributions of circRNAs. B, Classification of circRNAs. C, Distribution of exon composition. AG: abnormal group; NG: normal group

These circRNAs were widely scattered on almost all bovine chromosomes, and chromosome 1 was the most abundant, followed by chromosome X and 2 (Figure 1A). The properties of circRNAs contain classic, alter exon, intron, overlap exon, antisense, and intergenic. The compositional type of each sample is shown in Figure 1B. In total, the ratio of classic was the largest, exceeding 60% in each sample. CircRNAs transcribed from three exons (3‐exon circRNAs) were the most abundant circRNAs in all samples, followed by 2‐exon and 4‐exon circRNAs (Figure 1C).

### 3.2 Identification validation of differentially expressed circRNAs between AG and NG

**Figure 2:**
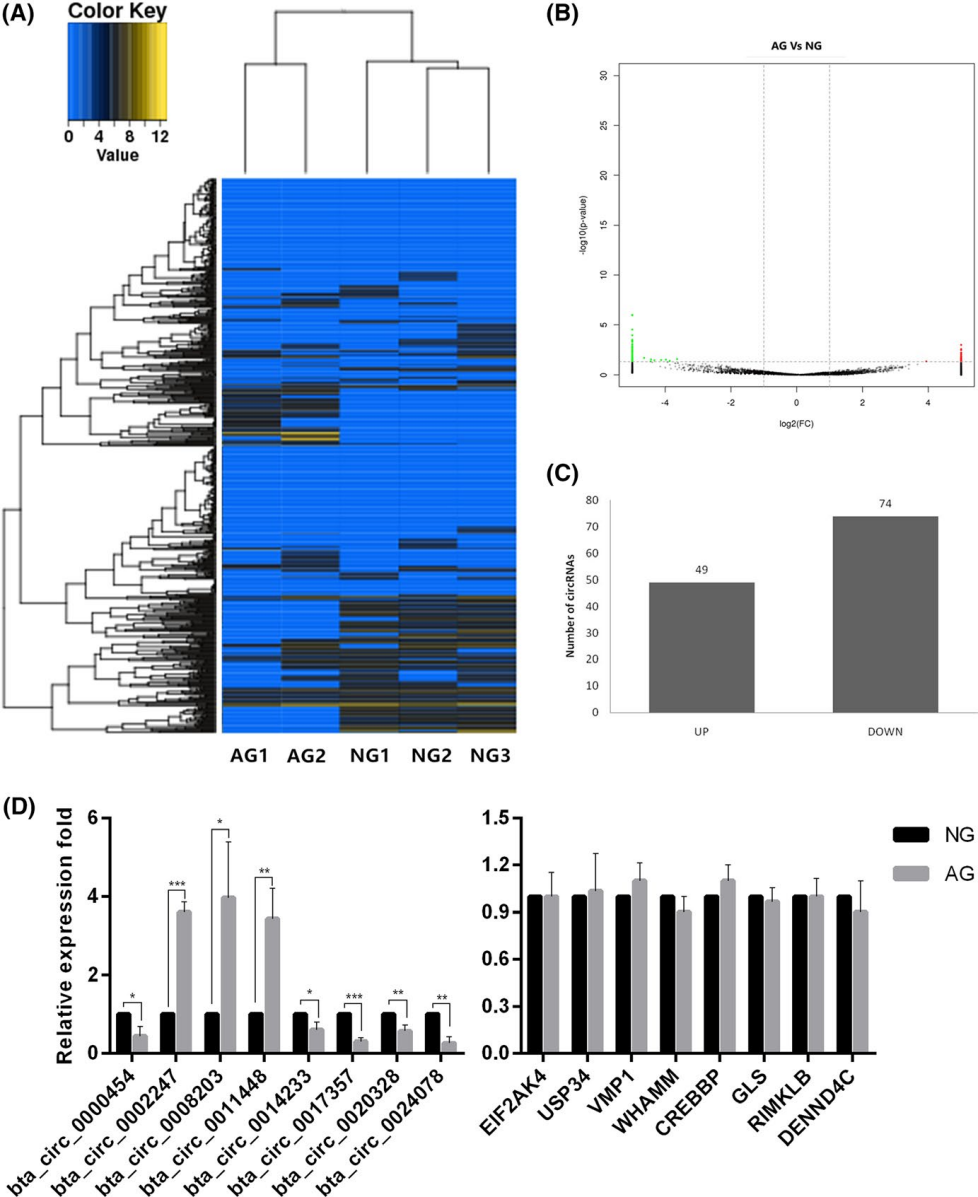
Differential expression of circRNAs between AG and NG A, Hierarchical cluster analysis of circRNAs. The color scale of the strips runs from blue (low relative expression) through black (medium relative expression) to yellow (high relative expression). B, Volcano plots visualize the differentially expressed (DE) circRNAs. The green and red plots represent the significantly DE circRNAs (FC ≥ 2.0, *P* value <0.05). C, Number of upregulated and downregulated circRNAs of DE circRNAs. D, Validation of DE circRNA expression level by qRT‐PCR. Eight DE circRNAs (left) and their host genes (right) were selected to validate the expression level (N = 3. *P < 0.05; **P < 0.01; ***P < 0.001)

Hierarchical cluster analysis was used to reveal the circRNA expression levels in AG and NG (Figure 2A), which showed that these levels were distinguishable between two groups. The significantly differentially expressed (DE) circRNAs between two groups were shown in the volcano plot (Figure 2B). In total, 123 circRNAs were identified as differentially expressed circRNAs by the filter criteria of fold change (FC) ≥2.0, *P* value <0.05. Among these, 49 circRNAs were upregulated, and 74 circRNAs were downregulated (Figure 2C). The differentially expressed circRNAs were listed in supplemental Table S2. The top 10 upregulated circRNAs were bta_circ_0012985, bta_circ_0013071, bta_circ_0013074, bta_circ_0016024, bta_circ_0013068, bta_circ_0008816, bta_circ_0012982, bta_circ_0013072, bta_circ_0019285, and bta_circ_0013067. The top 10 downregulated circRNAs were bta_circ_0024234, bta_circ_0017528, bta_circ_0008077, bta_circ_0003222, bta_circ_0007500, bta_circ_0020328, bta_circ_0011001, bta_circ_0016364, bta_circ_0008839, and bta_circ_0016049. Eight DE circRNAs and relative host genes were validated by qRT‐PCR. The results of DE circRNAs showed similar trend with sequencing result, while their host genes with no significant difference (Figure 2D).

### 3.3 Host gene enrichment of DE circRNAs and functional analysis

Through edge R analysis, 60 host genes of DE circRNAs were enriched (Table 1). Gene ontology (GO) analysis of these genes showed that there were two genes related to protein K48‐linked deubiquitination, two genes related to retromer complex, and two genes related to endoplasmic reticulum‐Golgi intermediate compartment membrane (Table 2). However, none of the suggested correlations was significant (*P* > 0.05). These result indicated that the DE circRNAs may play a role in placental protein metabolism and transport. Kyoto Encyclopedia of Genes and Genomes (KEGG) analysis showed that the predicted host genes were associated with virus invasion and amine acid metabolism (Table 3). Unfortunately, the result was also not significant (*P* > 0.05). Combined with these results, we surmised that DE circRNAs may affect placental protein metabolism.

**Table 1:** Host gene enrichment of differentially expressed circRNAs

CircRNA_ID	Host gene	Aberrant group		Normal group						
		AG1_CPM	AG2_CPM	NG1_CPM	NG2_CPM	NG3_CPM	AG_mean	NG_mean	log2(fc)	*P* value
bta_circ_0000010	SPATA7	0	0	77.95	234.05	148.25	0.001	153.417	17.2271	0.043291
bta_circ_0000454	EIF2AK4	0	0	116.92	306.06	164.72	0.001	195.9	17.5798	0.025803
bta_circ_0001735	RMDN2	779.12	355.49	0	108.02	0	567.305	36.0067	−3.9778	0.030563
bta_circ_0001852	PLEKHH2	467.47	0	0	0	0	233.735	0.001	−17.835	0.03198
bta_circ_0002247	USP34	272.69	71.1	0	0	0	171.895	0.001	−17.391	0.023302
bta_circ_0002631	ASAP2	0	0	97.43	126.03	164.72	0.001	129.393	16.9814	0.049523
bta_circ_0003287	ATP12A	233.74	284.39	0	36.01	0	259.065	12.0033	−4.4318	0.028896
bta_circ_0005735	TRIM6	155.82	106.65	0	0	0	131.235	0.001	−17.002	0.048234
bta_circ_0006537	PRDM2	662.25	0	0	0	0	331.125	0.001	−18.337	0.018117
bta_circ_0007503	OAS1Z	506.43	355.49	0	0	0	430.96	0.001	−18.717	0.000867
bta_circ_0007867	TANGO6	311.65	53.32	0	0	0	182.485	0.001	−17.477	0.021199
bta_circ_0008203	VMP1	311.65	35.55	0	0	0	173.6	0.001	−17.405	0.03001
bta_circ_0008450	ANKFY1	233.74	71.1	0	0	0	152.42	0.001	−17.218	0.034744
bta_circ_0008540	AKAP10	623.3	0	0	0	0	311.65	0.001	−18.25	0.019989
bta_circ_0008616	B4GALNT2	311.65	533.23	0	36.01	65.89	422.44	33.9667	−3.6366	0.02648
bta_circ_0009422	RYK	0	0	331.27	54.01	131.77	0.001	172.35	17.395	0.04259
bta_circ_0009546	BRWD1	0	0	233.84	306.06	148.25	0.001	229.383	17.8074	0.016827
bta_circ_0009559	BRWD1	389.56	53.32	0	0	0	221.44	0.001	−17.757	0.012467
bta_circ_0010335	PAK2	389.56	142.19	0	0	0	265.875	0.001	−18.02	0.004782
bta_circ_0010808	SLC38A9	194.78	106.65	0	0	0	150.715	0.001	−17.201	0.030874
bta_circ_0010882	PARP8	0	53.32	272.81	396.08	560.04	26.66	409.643	3.94162	0.046802
bta_circ_0011448	WHAMM	389.56	675.42	0	0	0	532.49	0.001	−19.022	0.000423
bta_circ_0011569	SCAPER	467.47	0	0	0	0	233.735	0.001	−17.835	0.032032
bta_circ_0012875	UBR2	0	0	311.78	54.01	164.72	0.001	176.837	17.4321	0.0407
bta_circ_0013512	OSBPL1A	0	0	350.75	198.04	98.83	0.001	215.873	17.7198	0.023113
bta_circ_0013840	WDR7	0	0	97.43	360.07	115.3	0.001	190.933	17.5427	0.032938
bta_circ_0013949	LITAF	233.74	248.84	0	36.01	0	241.29	12.0033	−4.3293	0.035077
bta_circ_0014233	CREBBP	0	0	253.32	342.07	263.55	0.001	286.313	18.1272	0.009391
bta_circ_0015895	RSF1	0	0	194.86	234.05	131.77	0.001	186.893	17.5119	0.025366
bta_circ_0016364	ACSL3	0	0	915.86	0	1729.53	0.001	881.797	19.7501	0.015514
bta_circ_0016481	DIS3L2	233.74	106.65	0	0	0	170.195	0.001	−17.377	0.020055
bta_circ_0016740	PLEKHB2	194.78	319.94	0	0	0	257.36	0.001	−17.973	0.004105
bta_circ_0017357	GLS	0	0	253.32	108.02	181.19	0.001	180.843	17.4644	0.02894
bta_circ_0017447	AOX1	0	0	175.38	90.02	164.72	0.001	143.373	17.1294	0.042112
bta_circ_0017528	RAPH1	0	0	545.62	126.03	691.81	0.001	454.487	18.7939	0.005775
bta_circ_0017631	FAAH	272.69	71.1	0	0	0	171.895	0.001	−17.391	0.023247
bta_circ_0017877	TRAF3IP1	0	0	175.38	288.06	115.3	0.001	192.913	17.5576	0.025511
bta_circ_0017956	UBAP2L	155.82	124.42	0	0	0	140.12	0.001	−17.096	0.03783
bta_circ_0017979	POGZ	155.82	142.19	0	0	0	149.005	0.001	−17.185	0.02946
bta_circ_0017986	MINDY1	506.43	0	0	0	0	253.215	0.001	−17.95	0.029053
bta_circ_0018723	DNAJC6	272.69	124.42	0	0	0	198.555	0.001	−17.599	0.011159
bta_circ_0018735	RAVER2	194.78	106.65	0	0	0	150.715	0.001	−17.201	0.030878
bta_circ_0019344	UBE3C	77.91	213.29	0	0	0	145.6	0.001	−17.152	0.032971
bta_circ_0019346	DNAJB6	0	0	155.89	126.03	181.19	0.001	154.37	17.236	0.037511
bta_circ_0020328	RIMKLB	0	0	0	828.17	1317.74	0.001	715.303	19.4482	0.018351
bta_circ_0020647	PLXNC1	545.38	0	0	0	0	272.69	0.001	−18.057	0.025205
bta_circ_0020821	PPHLN1	0	0	116.92	306.06	345.91	0.001	256.297	17.9675	0.015023
bta_circ_0021640	CDS1	0	0	233.84	90.02	197.66	0.001	173.84	17.4074	0.032148
bta_circ_0022053	CCSER1	0	0	272.81	108.02	280.02	0.001	220.283	17.749	0.019935
bta_circ_0022077	HERC6	0	0	77.95	216.04	164.72	0.001	152.903	17.2223	0.042512
bta_circ_0022579	SULT1E1	584.34	373.26	0	0	98.83	478.8	32.9433	−3.8614	0.040189
bta_circ_0023166	PRRC1	0	0	409.21	180.04	214.13	0.001	267.793	18.0308	0.012955
bta_circ_0023610	SSBP2	545.38	551	0	0	0	548.19	0.001	−19.064	0.000351
bta_circ_0024078	DENND4C	0	0	194.86	126.03	98.83	0.001	139.907	17.0941	0.046141
bta_circ_0024594	STC1	0	622.1	0	0	0	311.05	0.001	−18.247	0.015471
bta_circ_0024720	NTRK2	0	0	97.43	360.07	280.02	0.001	245.84	17.9074	0.018113
bta_circ_0025499	COQ3	311.65	213.29	0	0	0	262.47	0.001	−18.002	0.004004
bta_circ_0026690	SYTL4	0	0	97.43	126.03	181.19	0.001	134.883	17.0414	0.049087
bta_circ_0026843	CHM	0	0	155.89	180.04	230.6	0.001	188.843	17.5268	0.024553

**Table 2:** GO annotations of differentially expressed circRNA host genes

Category	Term	Count	*P* value	Genes
GOTERM_BP	Protein K48‐linked deubiquitination	2	0.060108	MINDY1; USP34
GOTERM_CC	Retromer complex	2	0.0515133	ANKFY1; DENND4C
GOTERM_CC	Endoplasmic reticulum‐Golgi intermediate compartment membrane	2	0.0544612	WHAMM; VMP1

**Table 3:** KEGG analysis of differentially expressed circRNA host genes

Category	Term	Count	*P* value	Genes
KEGG_PATHWAY	Influenza A	3	0.062348	CREBBP; EIF2AK4; OAS1Z
KEGG_PATHWAY	Herpes simplex infection	3	0.07349	CREBBP; EIF2AK4; OAS1Z
KEGG_PATHWAY	Alanine, aspartate, and glutamate metabolism	2	0.08218	GLS; RIMKLB

### 3.4 Prediction of differentially expressed circRNA‐miRNA interaction

CircRNAs act as miRNA sponges and exert their effects via the circRNA‐miRNA‐mRNA axis.^29^ Through miRanda based on the MREs, interaction between DE circRNAs and miRNAs was theoretically predicted. We found that 32 miRNAs could be paired with eight DE circRNAs (Table 4), with the criteria of a max score ≥140 and a max energy ≤−25 (a lower max energy is indicative of a stronger correlation). The result suggested that circRNAs may play a part in causing placental dysfunction via interaction with miRNAs.

**Table 4:** miRNA prediction which interact with differentially expressed circRNAs

CircRNA_ID	miRNA_Name
bta_circ_0006612	bta‐miR‐153; bta‐miR‐2325c; bta‐miR‐2340; bta‐miR‐2346; bta‐miR‐2897; bta‐miR‐383; bta‐miR‐544a; bta‐miR‐544b; bta‐miR‐545‐3p; bta‐miR‐574
bta_circ_0008203	bta‐miR‐200c
bta_circ_0008839	bta‐miR‐2285g; bta‐miR‐2285z; bta‐miR‐2399‐3p
bta_circ_0010876	bta‐miR‐1248
bta_circ_0013512	bta‐miR‐148b; bta‐miR‐152
bta_circ_0019285	bta‐miR‐145; bta‐miR‐181b; bta‐miR‐2285ad; bta‐miR‐2285n; bta‐miR‐2305; bta‐miR‐2411‐3p; bta‐miR‐342
bta_circ_0022053	bta‐miR‐29b; bta‐miR‐29c; bta‐miR‐29d‐3p
bta_circ_0026700	bta‐miR‐146b; bta‐miR‐2340; bta‐miR‐2355‐5p; bta‐miR‐544a; bta‐miR‐544b; bta‐miR‐574; bta‐miR‐6531; bta‐miR‐671

## 4 DISCUSSION

Compare with in vitro fertilized (IVF) embryo, SCNT embryo showed lower developmental efficiency. Due to the oocyte's microenvironment is suitable for gamete epigenetic reprogramming, somatic cell nucleus reprogramming in SCNT embryo is incomplete.^30^ Low birth rate and birth deficiency could be mainly ascribed to incomplete epigenetic reprogramming. Except fetus, extraembryonic tissue is also harmed by incorrect reprogramming, which leads to pregnancy failure. One symptom is cloned fetus aborted during late gestation, accompany with engorged uterus. We surmise that it is related to material transportation dysfunction. We observed that placental cotyledon present as different size in aberrant gestation recipient, which is uniform in normal gestation recipient. It may be compensatory hypertrophy. A equine clone research reported similar symptom,^5^ but no deep research. All of these symptoms were caused by abnormal gene expression. However, less of research focus on these abnormal SCNT fetal development which caused by placental dysfunction.

In this study, a total of 12 454 circRNAs were obtained. Yan et al^23^ obtained 48 270 circRNAs at human placental research. In other three reports of placental circRNA research, the number of sequenced circRNAs was similar as our study. In bovine, the study related to circRNA was not many. In circRNA expression study of bovine mammary glands, more than 6000 circRNAs were identified.^31^ In the research of genome‐wide analysis of circRNAs in bovine cumulus cells, 1706 circRNAs were identified.^32^ In another research, circular RNA profiling during myoblasts differentiation, 12 981 circRNAs were sequenced.^33^ In total, our data size was comparable with other circRNA research.

We predicted 60 target genes of differentially expressed circRNAs. The GO analysis indicated that MINDY1 and USP34 matched with protein K48‐linked deubiquitination, ANKFY1 and DENND4C matched with retromer complex, and WHAMM and VMP1 matched with endoplasmic reticulum‐Golgi intermediate compartment membrane. The results indicated aberrant bovine placenta may have dysfunctional endoplasmic reticulum‐Golgi intermediate material translation. KEGG analysis reflected that virus infection and alanine, aspartate and glutamate metabolism pathway‐related genes were involved. OAS1 has been found to be related to gestation.^34^, ^35^ EIF2AK4 belongs to a family of kinases that regulate angiogenesis in response to cellular stress, the mutation of which is likely to cause pulmonary capillary hemangiomatosis (PCH).^36^ CREBBP mutation is found accountable for a high incidence of preeclampsia.^37^ Glutamine plays a vital role in carbon and nitrogen metabolism of the fetus and exhibits the highest fetal‐maternal plasma ratio among all amino acids in pigs.^38^ These results indicated that differentially expressed circRNAs may have multiple effects in placental both structure and function.

CircRNAs could function with miRNAs and co‐regulate target genes' expression. We predicted 32 miRNAs which can pair with eight differentially expressed circRNAs. Among these miRNAs, miR‐145 was reported to be related to abnormal placental development in transgenic cloned cattle.^39^ Our results indicated that circRNAs may play a role in abnormal bovine fetus development in late gestation through interactions with miRNAs.

For it was one type of pregnancy familiar of bovine SCNT research and occurs randomly, the sample was not sufficient. CircRNAs as noncoding RNA need to contact with other RNA to act biological function. Further study is needed to explore the mechanism. Low birth rate of SCNT is a complicated question, and relative research should pay attention to placental dysfunction. Combined with multiple strategies, such as RNA expression, protein expression, histological and hormone analysis, the mechanism of bovine SCNT fetal abortion‐related placental dysfunction will be discovered. It is also helpful to improve the SCNT efficiency.

## 5 CONCLUSION

In this study, we acquired five circRNA expression profiles of SCNT bovine placentas (two abnormal and three normal) during late gestation. We identified 123 circRNAs were DE circRNAs between AG and NG. 60 target genes and 32 miRNAs were related to DE circRNAs. Through GO and KEGG analyses, we surmise that abnormal circRNA expression may play multiple roles in placental both structure and dysfunction. In the future, we would detect related mRNA and miRNA expression profiles to further explore its mechanism.
