# Supplementary material for: CircRNA expression profile of bovine placentas in late gestation with aberrant SCNT fetus
Source: J Clin Lab Anal. 2019 May 26;33(6):e22918. doi: 10.1002/jcla.22918 (PMC6642297; doi:10.1002/jcla.22918)
Supplement: Supplementary file 1 [file JCLA-33-e22918-s001.docx]

| **Supplemental Table S1. qRT-PCR Primers sequences** | | | | |  |  |  |
| --- | --- | --- | --- | --- | --- | --- | --- |
| DE circRNA | Sequence | | Length (bp) | Relative host gene | Sequence | | Length (bp) |
| bta_circ_0000454 | F: | TTGCTGGTGGCTCACTTTCA | 162 | EIF2AK4 | F: | CGGAGTCTGATCTCGTGGAC | 257 |
|  | R: | GTCAGGCTCTGGAGGCTTTT |  |  | R: | GGCAAGGGAGGTCTGAAGTC |  |
| bta_circ_0002247 | F: | CGCCATGTCAGTCTGTGACA | 146 | USP34 | F: | GCAGACCTGGTGGAGGTGTTA | 218 |
|  | R: | GATTGGTTGGCAGTGCAGAC |  |  | R: | CGGAGCACTTGAACTTGGGC |  |
| bta_circ_0008203 | F: | GTAAGCACAGCAAGCAGCAG | 240 | VMP1 | F: | GAGCGGCTCCTCAAGAGTTA | 297 |
|  | R: | TCTGTTGGGCTTGGAACAGG |  |  | R: | GCACAGCAAGCAGCAGTAAA |  |
| bta_circ_0011448 | F: | GTGTTGGCTTTTTCATGCCCT | 111 | WHAMM | F: | TCAACGAGCACATTCTGGCT | 280 |
|  | R: | ACCAGACAGGCTGAAGAAGC |  |  | R: | TTGTGCAGCCTGTCTACCAG |  |
| bta_circ_0014233 | F: | CACATTGCCTATTCCCGGGT | 262 | CREBBP | F: | ACCCCGAACAGAGTGCAATG | 255 |
|  | R: | AGCAAGCAGAGCATGGTCAA |  |  | R: | GCAATCTACCCTTCCATGGCT |  |
| bta_circ_0017357 | F: | CCAAACTAGGTAACAGACCCTGT | 142 | GLS | F: | TGTCACCTGAGTCAAATGAGGAC | 182 |
|  | R: | GCCCTGATTTGTGGGGTGTA |  |  | R: | GCACCCAAGGTTGAAAGCAC |  |
| bta_circ_0020328 | F: | GCAGCCACAGAGCTACACAT | 130 | RIMKLB | F: | TGAACCGACCTCAAGCCATC | 264 |
|  | R: | ACTGGACTTTAGGGCTGTGG |  |  | R: | GGTATGGAGCTTCATGGCGA |  |
| bta_circ_0024078 | F: | TCTCGGTCTTTGAGGTGAAGG | 230 | DENND4C | F: | CTGCCTTTTGGCCTGTTACC | 203 |
|  | R: | CCAATCACAGAGGCTCCTTCA |  |  | R: | GAGGGGATCCACAGGTCTTT |  |
|  |  |  |  | GAPDH | F: | ACCCAGAAGACTGTGGATGG | 125 |
|  |  |  |  |  | R: | TTCAGCTCAGGGATGACCTT |  |
