# Supplementary material for: CircRNA expression profile of bovine placentas in late gestation with aberrant SCNT fetus
Source: J Clin Lab Anal. 2019 May 26;33(6):e22918. doi: 10.1002/jcla.22918 (PMC6642297; doi:10.1002/jcla.22918)
Supplement: Supplementary file 2 [file JCLA-33-e22918-s002.docx]

| **Supplemental Table S2. Expression quantification of differentially expressed circRNAs in 5 samples.** | | | | | | | | |  |
| --- | --- | --- | --- | --- | --- | --- | --- | --- | --- |
| CircRNA_ID | Aberrant Group | | Nnormal Group | | | AG_mean | NG_mean | log2(fc) | P Value |
|  | AG1_CPM | AG2_CPM | NG1_CPM | NG2_CPM | NG3_CPM |  |  |  |  |
| bta_circ_0013071 | 2882.74 | 3661.51 | 0 | 0 | 0 | 3272.125 | 0.001 | -21.6418 | 9.94E-07 |
| bta_circ_0013074 | 3038.57 | 3270.47 | 0 | 0 | 0 | 3154.52 | 0.001 | -21.589 | 1.08E-06 |
| bta_circ_0013072 | 1207.64 | 1119.78 | 0 | 0 | 0 | 1163.71 | 0.001 | -20.1503 | 2.84E-05 |
| bta_circ_0013067 | 350.6 | 1777.43 | 0 | 0 | 0 | 1064.015 | 0.001 | -20.0211 | 0.00011 |
| bta_circ_0000822 | 740.16 | 444.36 | 0 | 0 | 0 | 592.26 | 0.001 | -19.1759 | 0.00031 |
| bta_circ_0023610 | 545.38 | 551 | 0 | 0 | 0 | 548.19 | 0.001 | -19.0643 | 0.00035 |
| bta_circ_0022835 | 1168.68 | 231.07 | 0 | 0 | 0 | 699.875 | 0.001 | -19.4167 | 0.00038 |
| bta_circ_0011448 | 389.56 | 675.42 | 0 | 0 | 0 | 532.49 | 0.001 | -19.0224 | 0.00042 |
| bta_circ_0012985 | 0 | 9722.54 | 0 | 0 | 0 | 4861.27 | 0.001 | -22.2129 | 0.00052 |
| bta_circ_0007503 | 506.43 | 355.49 | 0 | 0 | 0 | 430.96 | 0.001 | -18.7172 | 0.00087 |
| bta_circ_0016024 | 0 | 4976.8 | 0 | 0 | 0 | 2488.4 | 0.001 | -21.2468 | 0.00095 |
| bta_circ_0011001 | 0 | 0 | 798.94 | 1008.21 | 444.74 | 0.001 | 750.63 | 19.51774 | 0.00104 |
| bta_circ_0013068 | 0 | 3377.12 | 0 | 0 | 0 | 1688.56 | 0.001 | -20.6874 | 0.00141 |
| bta_circ_0008816 | 0 | 3306.02 | 0 | 0 | 0 | 1653.01 | 0.001 | -20.6567 | 0.00146 |
| bta_circ_0026481 | 934.94 | 88.87 | 0 | 0 | 0 | 511.905 | 0.001 | -18.9655 | 0.0016 |
| bta_circ_0012982 | 2999.61 | 0 | 0 | 0 | 0 | 1499.805 | 0.001 | -20.5163 | 0.00217 |
| bta_circ_0003222 | 0 | 0 | 487.16 | 288.06 | 856.53 | 0.001 | 543.917 | 19.05303 | 0.00253 |
| bta_circ_0019285 | 2298.4 | 0 | 0 | 0 | 0 | 1149.2 | 0.001 | -20.1322 | 0.00292 |
| bta_circ_0016068 | 194.78 | 391.03 | 0 | 0 | 0 | 292.905 | 0.001 | -18.1601 | 0.00304 |
| bta_circ_0008077 | 0 | 0 | 331.27 | 666.14 | 395.32 | 0.001 | 464.243 | 18.82452 | 0.00317 |
| bta_circ_0007500 | 0 | 0 | 214.35 | 1170.24 | 428.27 | 0.001 | 604.287 | 19.20487 | 0.00323 |
| bta_circ_0006612 | 272.69 | 266.61 | 0 | 0 | 0 | 269.65 | 0.001 | -18.0407 | 0.00372 |
| bta_circ_0025499 | 311.65 | 213.29 | 0 | 0 | 0 | 262.47 | 0.001 | -18.0018 | 0.004 |
| bta_circ_0016740 | 194.78 | 319.94 | 0 | 0 | 0 | 257.36 | 0.001 | -17.9734 | 0.0041 |
| bta_circ_0013087 | 545.38 | 88.87 | 0 | 0 | 0 | 317.125 | 0.001 | -18.2747 | 0.00451 |
| bta_circ_0010335 | 389.56 | 142.19 | 0 | 0 | 0 | 265.875 | 0.001 | -18.0204 | 0.00478 |
| bta_circ_0002865 | 389.56 | 142.19 | 0 | 0 | 0 | 265.875 | 0.001 | -18.0204 | 0.00479 |
| bta_circ_0023167 | 350.6 | 159.97 | 0 | 0 | 0 | 255.285 | 0.001 | -17.9617 | 0.00501 |
| bta_circ_0013065 | 0 | 1244.2 | 0 | 0 | 0 | 622.1 | 0.001 | -19.2468 | 0.0051 |
| bta_circ_0026483 | 1480.33 | 0 | 0 | 0 | 0 | 740.165 | 0.001 | -19.4975 | 0.00511 |
| bta_circ_0017528 | 0 | 0 | 545.62 | 126.03 | 691.81 | 0.001 | 454.487 | 18.79388 | 0.00578 |
| bta_circ_0007213 | 0 | 0 | 311.78 | 342.07 | 362.38 | 0.001 | 338.743 | 18.36983 | 0.00578 |
| bta_circ_0026390 | 350.6 | 124.42 | 0 | 0 | 0 | 237.51 | 0.001 | -17.8576 | 0.00695 |
| bta_circ_0013080 | 194.78 | 213.29 | 0 | 0 | 0 | 204.035 | 0.001 | -17.6385 | 0.00804 |
| bta_circ_0008133 | 0 | 0 | 311.78 | 486.1 | 181.19 | 0.001 | 326.357 | 18.31609 | 0.00806 |
| bta_circ_0014233 | 0 | 0 | 253.32 | 342.07 | 263.55 | 0.001 | 286.313 | 18.12724 | 0.00939 |
| bta_circ_0018723 | 272.69 | 124.42 | 0 | 0 | 0 | 198.555 | 0.001 | -17.5992 | 0.01116 |
| bta_circ_0026566 | 0 | 728.75 | 0 | 0 | 0 | 364.375 | 0.001 | -18.4751 | 0.01215 |
| bta_circ_0009559 | 389.56 | 53.32 | 0 | 0 | 0 | 221.44 | 0.001 | -17.7566 | 0.01247 |
| bta_circ_0026637 | 0 | 710.97 | 0 | 0 | 0 | 355.485 | 0.001 | -18.4394 | 0.01261 |
| bta_circ_0023166 | 0 | 0 | 409.21 | 180.04 | 214.13 | 0.001 | 267.793 | 18.03076 | 0.01296 |
| bta_circ_0020821 | 0 | 0 | 116.92 | 306.06 | 345.91 | 0.001 | 256.297 | 17.96746 | 0.01502 |
| bta_circ_0024594 | 0 | 622.1 | 0 | 0 | 0 | 311.05 | 0.001 | -18.2468 | 0.01547 |
| bta_circ_0016364 | 0 | 0 | 915.86 | 0 | 1729.53 | 0.001 | 881.797 | 19.75009 | 0.01551 |
| bta_circ_0009546 | 0 | 0 | 233.84 | 306.06 | 148.25 | 0.001 | 229.383 | 17.8074 | 0.01683 |
| bta_circ_0000738 | 0 | 0 | 311.78 | 180.04 | 181.19 | 0.001 | 224.337 | 17.77531 | 0.01767 |
| bta_circ_0006894 | 662.25 | 0 | 0 | 0 | 0 | 331.125 | 0.001 | -18.337 | 0.01811 |
| bta_circ_0024720 | 0 | 0 | 97.43 | 360.07 | 280.02 | 0.001 | 245.84 | 17.90736 | 0.01811 |
| bta_circ_0006537 | 662.25 | 0 | 0 | 0 | 0 | 331.125 | 0.001 | -18.337 | 0.01812 |
| bta_circ_0013069 | 0 | 568.78 | 0 | 0 | 0 | 284.39 | 0.001 | -18.1175 | 0.0183 |
| bta_circ_0020328 | 0 | 0 | 0 | 828.17 | 1317.74 | 0.001 | 715.303 | 19.4482 | 0.01835 |
| bta_circ_0026484 | 116.87 | 213.29 | 0 | 0 | 0 | 165.08 | 0.001 | -17.3328 | 0.01937 |
| bta_circ_0022053 | 0 | 0 | 272.81 | 108.02 | 280.02 | 0.001 | 220.283 | 17.749 | 0.01993 |
| bta_circ_0015292 | 623.3 | 0 | 0 | 0 | 0 | 311.65 | 0.001 | -18.2496 | 0.01996 |
| bta_circ_0008540 | 623.3 | 0 | 0 | 0 | 0 | 311.65 | 0.001 | -18.2496 | 0.01999 |
| bta_circ_0005992 | 623.3 | 0 | 0 | 0 | 0 | 311.65 | 0.001 | -18.2496 | 0.02 |
| bta_circ_0016481 | 233.74 | 106.65 | 0 | 0 | 0 | 170.195 | 0.001 | -17.3768 | 0.02005 |
| bta_circ_0000840 | 1441.37 | 177.74 | 97.43 | 0 | 0 | 809.555 | 32.4767 | -4.63965 | 0.02014 |
| bta_circ_0007867 | 311.65 | 53.32 | 0 | 0 | 0 | 182.485 | 0.001 | -17.4774 | 0.0212 |
| bta_circ_0005485 | 0 | 0 | 253.32 | 90.02 | 329.44 | 0.001 | 224.26 | 17.77481 | 0.02162 |
| bta_circ_0013512 | 0 | 0 | 350.75 | 198.04 | 98.83 | 0.001 | 215.873 | 17.71983 | 0.02311 |
| bta_circ_0017631 | 272.69 | 71.1 | 0 | 0 | 0 | 171.895 | 0.001 | -17.3912 | 0.02325 |
| bta_circ_0002247 | 272.69 | 71.1 | 0 | 0 | 0 | 171.895 | 0.001 | -17.3912 | 0.0233 |
| bta_circ_0022234 | 0 | 0 | 214.35 | 108.02 | 280.02 | 0.001 | 200.797 | 17.61538 | 0.02367 |
| bta_circ_0012668 | 0 | 0 | 194.86 | 162.03 | 214.13 | 0.001 | 190.34 | 17.53822 | 0.02403 |
| bta_circ_0026843 | 0 | 0 | 155.89 | 180.04 | 230.6 | 0.001 | 188.843 | 17.52683 | 0.02455 |
| bta_circ_0020647 | 545.38 | 0 | 0 | 0 | 0 | 272.69 | 0.001 | -18.0569 | 0.02521 |
| bta_circ_0015895 | 0 | 0 | 194.86 | 234.05 | 131.77 | 0.001 | 186.893 | 17.51186 | 0.02537 |
| bta_circ_0017877 | 0 | 0 | 175.38 | 288.06 | 115.3 | 0.001 | 192.913 | 17.55759 | 0.02551 |
| bta_circ_0000454 | 0 | 0 | 116.92 | 306.06 | 164.72 | 0.001 | 195.9 | 17.57976 | 0.0258 |
| bta_circ_0008616 | 311.65 | 533.23 | 0 | 36.01 | 65.89 | 422.44 | 33.9667 | -3.63655 | 0.02648 |
| bta_circ_0024234 | 0 | 0 | 701.51 | 0 | 625.93 | 0.001 | 442.48 | 18.75525 | 0.02871 |
| bta_circ_0003287 | 233.74 | 284.39 | 0 | 36.01 | 0 | 259.065 | 12.0033 | -4.43181 | 0.0289 |
| bta_circ_0017357 | 0 | 0 | 253.32 | 108.02 | 181.19 | 0.001 | 180.843 | 17.46438 | 0.02894 |
| bta_circ_0017986 | 506.43 | 0 | 0 | 0 | 0 | 253.215 | 0.001 | -17.95 | 0.02905 |
| bta_circ_0013242 | 506.43 | 0 | 0 | 0 | 0 | 253.215 | 0.001 | -17.95 | 0.02908 |
| bta_circ_0017979 | 155.82 | 142.19 | 0 | 0 | 0 | 149.005 | 0.001 | -17.185 | 0.02946 |
| bta_circ_0008203 | 311.65 | 35.55 | 0 | 0 | 0 | 173.6 | 0.001 | -17.4054 | 0.03001 |
| bta_circ_0013066 | 116.87 | 177.74 | 0 | 0 | 0 | 147.305 | 0.001 | -17.1684 | 0.03005 |
| bta_circ_0001735 | 779.12 | 355.49 | 0 | 108.02 | 0 | 567.305 | 36.0067 | -3.97779 | 0.03056 |
| bta_circ_0025923 | 272.69 | 53.32 | 0 | 0 | 0 | 163.005 | 0.001 | -17.3146 | 0.03083 |
| bta_circ_0010808 | 194.78 | 106.65 | 0 | 0 | 0 | 150.715 | 0.001 | -17.2015 | 0.03087 |
| bta_circ_0018735 | 194.78 | 106.65 | 0 | 0 | 0 | 150.715 | 0.001 | -17.2015 | 0.03088 |
| bta_circ_0001852 | 467.47 | 0 | 0 | 0 | 0 | 233.735 | 0.001 | -17.8345 | 0.03198 |
| bta_circ_0011569 | 467.47 | 0 | 0 | 0 | 0 | 233.735 | 0.001 | -17.8345 | 0.03203 |
| bta_circ_0021640 | 0 | 0 | 233.84 | 90.02 | 197.66 | 0.001 | 173.84 | 17.4074 | 0.03215 |
| bta_circ_0013840 | 0 | 0 | 97.43 | 360.07 | 115.3 | 0.001 | 190.933 | 17.54271 | 0.03294 |
| bta_circ_0019344 | 77.91 | 213.29 | 0 | 0 | 0 | 145.6 | 0.001 | -17.1517 | 0.03297 |
| bta_circ_0008633 | 0 | 0 | 175.38 | 108.02 | 214.13 | 0.001 | 165.843 | 17.33946 | 0.03313 |
| bta_circ_0007465 | 779.12 | 177.74 | 0 | 0 | 82.36 | 478.43 | 27.4533 | -4.12325 | 0.03316 |
| bta_circ_0010876 | 0 | 0 | 97.43 | 198.04 | 197.66 | 0.001 | 164.377 | 17.32665 | 0.03379 |
| bta_circ_0008450 | 233.74 | 71.1 | 0 | 0 | 0 | 152.42 | 0.001 | -17.2177 | 0.03474 |
| bta_circ_0016049 | 0 | 0 | 16115.2 | 0 | 0 | 0.001 | 5371.73 | 22.35696 | 0.03496 |
| bta_circ_0013949 | 233.74 | 248.84 | 0 | 36.01 | 0 | 241.29 | 12.0033 | -4.32926 | 0.03508 |
| bta_circ_0017608 | 0 | 0 | 97.43 | 180.04 | 197.66 | 0.001 | 158.377 | 17.273 | 0.03661 |
| bta_circ_0024766 | 0 | 0 | 194.86 | 180.04 | 98.83 | 0.001 | 157.91 | 17.26874 | 0.03662 |
| bta_circ_0019346 | 0 | 0 | 155.89 | 126.03 | 181.19 | 0.001 | 154.37 | 17.23603 | 0.03751 |
| bta_circ_0014576 | 428.52 | 0 | 0 | 0 | 0 | 214.26 | 0.001 | -17.709 | 0.03772 |
| bta_circ_0004372 | 428.52 | 0 | 0 | 0 | 0 | 214.26 | 0.001 | -17.709 | 0.03775 |
| bta_circ_0026700 | 428.52 | 0 | 0 | 0 | 0 | 214.26 | 0.001 | -17.709 | 0.03776 |
| bta_circ_0017956 | 155.82 | 124.42 | 0 | 0 | 0 | 140.12 | 0.001 | -17.0963 | 0.03783 |
| bta_circ_0014982 | 0 | 0 | 155.89 | 90.02 | 230.6 | 0.001 | 158.837 | 17.27718 | 0.03821 |
| bta_circ_0008485 | 0 | 373.26 | 0 | 0 | 0 | 186.63 | 0.001 | -17.5098 | 0.03894 |
| bta_circ_0000884 | 0 | 0 | 116.92 | 126.03 | 214.13 | 0.001 | 152.36 | 17.21712 | 0.03972 |
| bta_circ_0022579 | 584.34 | 373.26 | 0 | 0 | 98.83 | 478.8 | 32.9433 | -3.86136 | 0.04019 |
| bta_circ_0012875 | 0 | 0 | 311.78 | 54.01 | 164.72 | 0.001 | 176.837 | 17.43206 | 0.0407 |
| bta_circ_0025131 | 0 | 0 | 253.32 | 54.01 | 197.66 | 0.001 | 168.33 | 17.36093 | 0.04154 |
| bta_circ_0017447 | 0 | 0 | 175.38 | 90.02 | 164.72 | 0.001 | 143.373 | 17.12942 | 0.04211 |
| bta_circ_0022077 | 0 | 0 | 77.95 | 216.04 | 164.72 | 0.001 | 152.903 | 17.22226 | 0.04251 |
| bta_circ_0009422 | 0 | 0 | 331.27 | 54.01 | 131.77 | 0.001 | 172.35 | 17.39498 | 0.04259 |
| bta_circ_0000010 | 0 | 0 | 77.95 | 234.05 | 148.25 | 0.001 | 153.417 | 17.2271 | 0.04329 |
| bta_circ_0024475 | 0 | 0 | 272.81 | 0 | 691.81 | 0.001 | 321.54 | 18.29464 | 0.04444 |
| bta_circ_0017895 | 0 | 355.49 | 0 | 0 | 0 | 177.745 | 0.001 | -17.4394 | 0.04446 |
| bta_circ_0008839 | 0 | 0 | 0 | 7039.46 | 0 | 0.001 | 2346.49 | 21.16207 | 0.04476 |
| bta_circ_0024078 | 0 | 0 | 194.86 | 126.03 | 98.83 | 0.001 | 139.907 | 17.09411 | 0.04614 |
| bta_circ_0010882 | 0 | 53.32 | 272.81 | 396.08 | 560.04 | 26.66 | 409.643 | 3.94162 | 0.0468 |
| bta_circ_0012975 | 11453.06 | 1279.75 | 876.89 | 0 | 0 | 6366.405 | 292.297 | -4.44497 | 0.04739 |
| bta_circ_0000804 | 0 | 0 | 97.43 | 270.06 | 82.36 | 0.001 | 149.95 | 17.19412 | 0.04743 |
| bta_circ_0005735 | 155.82 | 106.65 | 0 | 0 | 0 | 131.235 | 0.001 | -17.0018 | 0.04823 |
| bta_circ_0013064 | 155.82 | 106.65 | 0 | 0 | 0 | 131.235 | 0.001 | -17.0018 | 0.04824 |
| bta_circ_0008775 | 389.56 | 0 | 0 | 0 | 0 | 194.78 | 0.001 | -17.5715 | 0.04886 |
| bta_circ_0026690 | 0 | 0 | 97.43 | 126.03 | 181.19 | 0.001 | 134.883 | 17.04135 | 0.04909 |
| bta_circ_0002631 | 0 | 0 | 97.43 | 126.03 | 164.72 | 0.001 | 129.393 | 16.9814 | 0.04952 |
